# Concurrent regulation of LKB1 and CaMKK2 in the activation of AMPK in castrate-resistant prostate cancer by a well-defined polyherbal mixture with anticancer properties

**DOI:** 10.1186/s12906-018-2255-0

**Published:** 2018-06-18

**Authors:** Amber F. MacDonald, Ahmed Bettaieb, Dallas R. Donohoe, Dina S. Alani, Anna Han, Yi Zhao, Jay Whelan

**Affiliations:** 1 Department of Nutrition, University of Tennessee, 1215 West Cumberland Avenue, 229 Jessie Harris Building, Knoxville, TN 37996 USA; 2 Tennessee Agricultural Experiment Station, University of Tennessee, Knoxville, TN 37996 USA; 3 Department of Nutrition, Laboratory for Cancer Research, University of Tennessee, 1215 West Cumberland Avenue, Room 229 Jessie Harris Building, Knoxville, TN 37996-1920 USA; 4 Present addresses: Kellogg Eye Center, University of Michigan, 1000 Wall St, Ann Arbor, MI 48105 USA; 5 Present addresses: Department of Cancer Biology, Thomas Jefferson University, 233 S.10th Street, Philadelphia, PA 19107 USA

**Keywords:** Zyflamend, AMPK, LKB1, CaMKK2, CWR22Rv1, HeLa, DAPK, PKC-zeta, Prostate cancer, Castrate-resistant

## Abstract

**Background:**

Zyflamend, a blend of herbal extracts, effectively inhibits tumor growth using preclinical models of castrate-resistant prostate cancer mediated in part by 5′-adenosine monophosphate-activated protein kinase (AMPK), a master energy sensor of the cell. Clinically, treatment with Zyflamend and/or metformin (activators of AMPK) had benefits in castrate-resistant prostate cancer patients who no longer responded to treatment. Two predominant upstream kinases are known to activate AMPK: liver kinase B1 (LKB1), a tumor suppressor, and calcium-calmodulin kinase kinase-2 (CaMKK2), a tumor promotor over-expressed in many cancers. The objective was to interrogate how Zyflamend activates AMPK by determining the roles of LKB1 and CaMKK2.

**Methods:**

AMPK activation was determined in CWR22Rv1 cells treated with a variety of inhibitors of LKB1 and CaMKK2 in the presence and absence of Zyflamend, and in LKB1-null HeLa cells that constitutively express CaMKK2, following transfection with wild type LKB1 or catalytically-dead mutants. Upstream regulation by Zyflamend of LKB1 and CaMKK2 was investigated targeting protein kinase C-zeta (PKCζ) and death-associated protein kinase (DAPK), respectively.

**Results:**

Zyflamend’s activation of AMPK appears to be LKB1 dependent, while simultaneously inhibiting CaMKK2 activity. Zyflamend failed to rescue the activation of AMPK in the presence of pharmacological and molecular inhibitors of LKB1, an effect not observed in the presence of inhibitors of CaMKK2. Using LKB1-null and catalytically-dead LKB1-transfected HeLa cells that constitutively express CaMKK2, ionomycin (activator of CaMKK2) increased phosphorylation of AMPK, but Zyflamend only had an effect in cells transfected with wild type LKB1. Zyflamend appears to inhibit CaMKK2 by DAPK-mediated phosphorylation of CaMKK2 at Ser511, an effect prevented by a DAPK inhibitor. Alternatively, Zyflamend mediates LKB1 activation via increased phosphorylation of PKCζ, where it induced translocation of PKCζ and LKB1 to their respective active compartments in HeLa cells following treatment. Altering the catalytic activity of LKB1 did not alter this translocation.

**Discussion:**

Zyflamend’s activation of AMPK is mediated by LKB1, possibly via PKCζ, but independent of CaMKK2 by a mechanism that appears to involve DAPK.

**Conclusions:**

Therefore, this is the first evidence that natural products simultaneously and antithetically regulate upstream kinases, known to be involved in cancer, via the activation of AMPK.

**Electronic supplementary material:**

The online version of this article (10.1186/s12906-018-2255-0) contains supplementary material, which is available to authorized users.

## Background

Prostate cancer is the second leading cause of death for men in the United States [1]. While early stages of the disease are treatable, with 5-year survival rates near 100%, prognosis for advanced forms are less promising [2]. Initially, prostate cancer cells rely on androgens for growth, and chemically-mediated deprivation (hormone deprivation therapy) is a common therapy that results in cancer regression [3]. Relapse in the absence of androgens (castrate-resistant prostate cancer) is inevitable for most individuals and is associated with increased expression and activation of the androgen receptor, a major determinant in survival [4, 5]. Due to the poor prognosis of castrate-resistant prostate cancer, concomitant use of natural products to enhance effectiveness is being explored clinically and experimentally [3, 6–10].

**Fig. 1:**
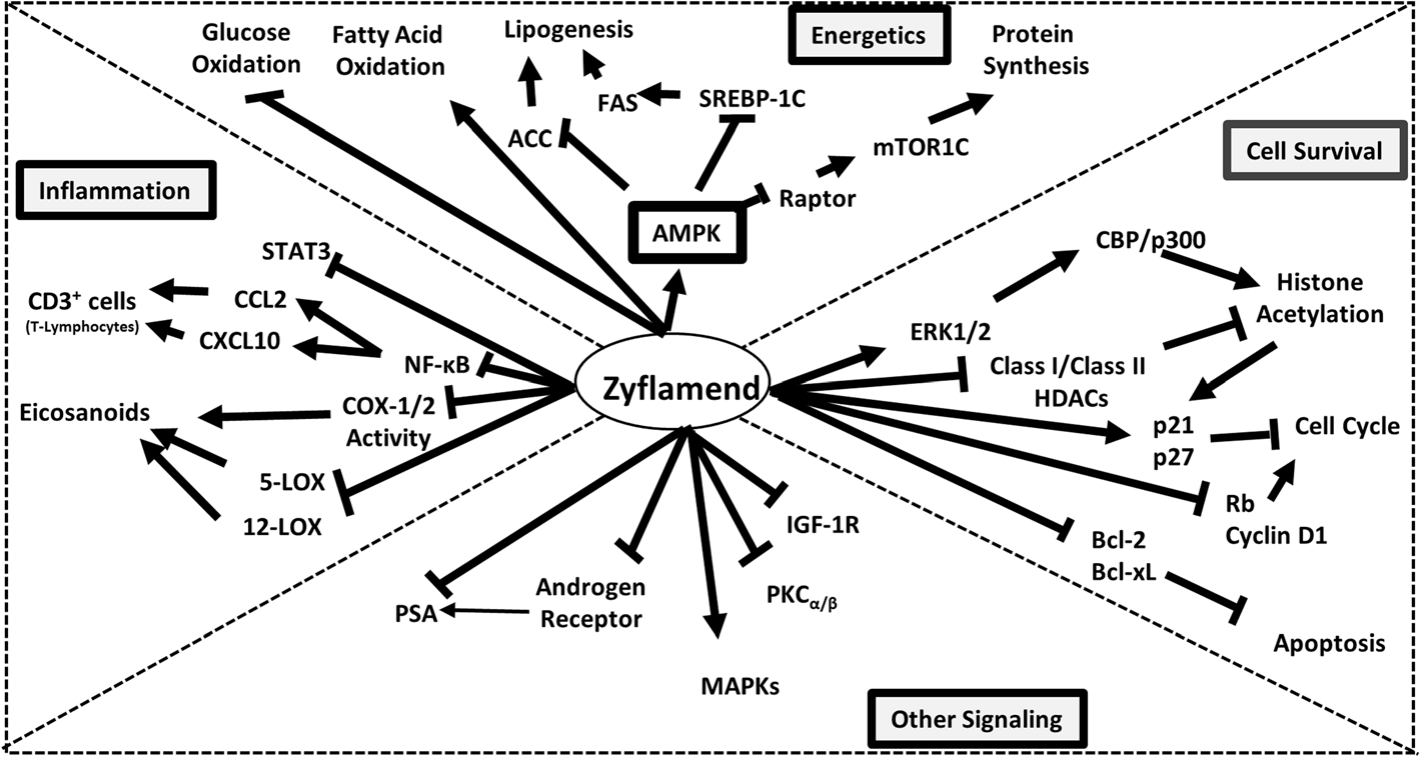
Summary of the effects of Zyflamend on prostate cancer (with permission from reference [3])

Zyflamend (New Chapter, Inc. Brattleboro, VT) is a poly-herbal supplement derived from the extracts of ten different herbs: rosemary, turmeric, holy basil, ginger, green tea, hu zhang, barberry, oregano, Chinese goldthread, and baikal skullcap. Most research using Zyflamend has focused its effects on a variety of cancer models, including oral [11], mammary [12], bone [13], pancreas [14, 15], skin [11, 16], colorectal [15], with an emphasis on prostate [6–9, 17–21], and its beneficial effects appear to be related to the synergy of action of its components [22]. The effects of Zyflamend and its mechanisms on prostate cancer has been reviewed elsewhere and can be summarized in Fig. 1 [3]. Zyflamend inhibits signaling pathways of inflammation, affects cell survival by enhancing apoptotic and tumor suppressor genes, epigenetically modifies histones, down regulates the androgen receptor and influences the energetics of the cell. The latter pathways are critically important in cancer as rapidly dividing cells rely on the increased synthesis of macromolecules (lipids, proteins, nucleotides, etc) (as reviewed in [23].

5′-adenosine monophosphate-activated protein kinase (AMPK) is a key regulator of energy in the cell and responds to deficits in adenosine triphosphate (ATP). The protein contains a catalytic subunit (α-subunit), and two regulatory subunits, β and γ-subunits. Under conditions of energy stress the following occurs, *(i)* increased levels of AMP or ADP bind to the γ-subunit causing allosteric activation of the protein (ATP is a competitive inhibitor), *(ii)* increased affinity for upstream kinases that target phosphorylation at Thr172 of the α-subunit (increasing catalytic activity > 100 fold), and *(iii)* reduced affinity for phosphatases that are involved in dephosphorylation at Thr172 [24]. When activated, AMPK is instrumental in inhibiting anabolic pathways that consume ATP, such as lipogenesis and protein synthesis, and enhances catabolic pathways that generate ATP, such as fatty acid oxidation [23].

Clinically, treatment with Zyflamend and/or metformin (activator of AMPK) had benefits in castrate-resistant prostate cancer patients who no longer responded to a variety of treatments (e.g., hormone ablation, immune-, chemo-, and radiation therapy). Recently, it was determined that tumor suppressor properties of Zyflamend are associated with the activation of AMPK and its downstream signaling, where siRNA knockdown, pharmacological inhibition and over expression of AMPK confirmed Zyflamend’s involvement [10]. This involves inhibiting the mammalian target of rapamycin complex-1 (mTORC1) and protein synthesis, lipogenesis by targeting the expression of *(i)* fatty acid synthase, *(ii)* the sterol regulatory element-binding transcription factor-1c, and *(iii)* inhibiting the activity of acetyl CoA carboxylase (ACC). What is not known is how Zyflamend upregulates AMPK. Four kinases have been identified that activate AMPK at Thr172, liver kinases B1 (LKB1), calcium-calmodulin kinase kinase-2 (CaMKK2), transforming growth factor-β activated protein kinase-1 (TAK1) and mixed lineage kinase 3 (MLK3) [25–28]. LKB1 and CaMKK2 are important in a number of cancers, including castrate-resistant prostate cancer (as reviewed in [29]), while the involvement of TAK1 and MLK3 has yet to be determined. LKB1 responds to increases in AMP and ADP, while increases in intracellular calcium is needed for activation of CaMKK2 without requiring elevation in AMP or ADP.

Interestingly, while both LKB1 and CaMKK2 are involved in activating AMPK, their effects on cancer appear to be quite different. LKB1 has anticancer properties because its mutation/deletion is associated with a variety of cancers [30]. CaMKK2, on the other hand, is overexpressed in a number of cancers, including castrate-resistant prostate cancer [31, 32]. Therefore, the overall objective of this paper was to interrogate how Zyflamend activates AMPK in a model of castrate-resistant prostate cancer and the roles LKB1 and CaMKK2 play in that activation.

The major findings of this research revealed that although LKB1 and CaMMK2 are upstream kinases that can activate AMPK, Zyflamend inhibits CaMMK2, a protein overexpressed in many cancers, while simultaneously upregulates LKB1, a reported tumor suppressor. This is the first report linking the simultaneous antagonistic regulation of these two proteins. Upstream regulation of CaMKK2 appears to be mediated by the antitumorigenic death associated protein kinase (DAPK) [33–36], not epigenetically, but via phosphorylation at Ser511 [37]. This is only the second paper to link DAPK activity with the negative regulation of CaMKK2 via phosphorylation at Ser511, and the only one involving cancer cells.

## Methods

Zyflamend (New Chapter, Inc. Brattleboro, VT), purchased from Earth Fare Supermarket (Knoxville, TN), is composed of extracts from the following herbs (*w*/w): rosemary (*Rosmarinus officinalis* 19.2%), turmeric (*Curcuma longa* 14.1%), holy basil (*Ocimum sanctum* 12.8%), ginger (*Zingiber officinale* 12.8%), green tea (*Camellia sinensis* 12.8%), hu zhang (*Polygonum cuspidatum* 10.2%), barberry (*Berberis vulgaris* 5.1%), oregano (*Origanum vulgare* 5.1%), Chinese goldthread (*Coptis chinensis* 5.1%), and baikal skullcap (*Scutellaria baicalensis* 2.5%). Detailed description and characterization of the preparation of Zyflamend and quality assurance of the mixture has been described previously in detail [7]. This description includes rigorously generated verifiable quality control of its constituents via multiple independent laboratories whose biological effects have been duplicated using different lots, at different times, under different experimental conditions, in different laboratories across the United States.

Dulbecco’s Modified Eagle Medium (DMEM), G418, penicillin/streptomycin, puromycin, fetal bovine serum (FBS) and trypsin were purchased from Invitrogen (Carlsbad, CA). Cloning vectors were purchased from Addgene (Cambridge, MA). Antibodies for PKC-zeta (PKCζ), LKB1, phospho-LKB1, green fluorescent protein (GFP), Histone B, Flag, and Tubulin were from Santa Cruz Biotechnology (Santa Cruz, CA). AMPK and phospho-AMPK were from Cell Signaling Technology (Beverly, MA). The following chemical reagents were purchased: 5-aminoimidazole-4-carboxamide ribonucleotide (AICAR) (AdipoGen Life Sciences, San Diego, CA); 1,2-bis(*o*-aminophenoxy)ethane-*N*,*N*,*N′*,*N′*-tetraacetic acid acetoxymethyl ester (BAPTA-AM) and ethylene glycol-bis(β-aminoethyl ether)-N,N,N′,N′-tetraacetic acid (EGTA) (Thermo Scientific, Rockville, IL); STO-609, radicicol, and PKCζ Pseudo-substrate Inhibitor (Santa Cruz Biotechnology, Dallas, TX); ionomycin (Sigma-Aldrich, St. Louis, MO); and Death Associated Protein Kinase Inhibitor (DAPKi) (Merck Millipore, Billerica, MA).

### Cell culture

CWR22Rv1 cells (American Type Culture Collection, Rockville, MD), a human-derived castrate-resistant prostate cancer cell line, were cultured in RPMI 1640 media, supplemented with 10% FBS. To mimic an androgen-depleted state, the cells were incubated overnight with 0.5% FBS. HeLa cells (ATCC, Rockville, MD), a human-derived cervical cancer cell line that do not express LKB1, and HCT116 cells (ATCC, Rockville, MD), a human derived colorectal cancer cell line, were cultured in DMEM media supplemented with 10% FBS and 25 mM glucose. All cells were incubated under an atmosphere of 5% CO_2_, at 37 °C. For activation of AMPK via LKB1-dependent or CaMKK2-dependent pathways, cells were treated with AICAR (a cell permeable analog of AMP) (1 mM, 1 h) or ionomycin (calcium ionophore) (1 μM, 1 h), respectively. For experiments using inhibitors of CaMKK2, cells were pre-treated with the selective CaMKK2 inhibitor STO-609 (10 μM, 30 min) or the calcium chelators BAPTA-AM (30 μM, 30 min) or EGTA (2 mM, 30 min). For inhibition of LKB1 or DAPK, cells were pre-treated with radicicol (5 μM, 24 h) or DAPKi (20 μM, 24 h), respectively. For inhibition of PKCζ, cells were pre-treated with the selective PKCζ pseudo-substrate inhibitor (5 μM, 30 min). For all experiments involving Zyflamend, cells were treated with Zyflamend at 200 μg/mL for 30 min unless otherwise indicated.

### Down regulation of LKB1 by small interfering RNA

CWR22Rv1 cells were seeded in RPMI medium containing 10% FBS and incubated overnight before media was replaced with RNA transfection medium containing 0.5% FBS. Cells were transfected with 20 nmol of siRNA targeting LKB1 (Thermo Scientific/Dharmacon #L-005035-00) and a siRNA non-targeting control (Thermo Scientific/Dharmacon #D-001810-10-05). Western blot analysis confirmed the efficiency of knockdown to be 76% 48 h after transfection, at which time cells were treated with a vehicle or Zyflamend (200 μg/mL) for 30 min.

### Overexpression of LKB1 in HeLa cells

Human WT or catalytically dead (KD) mutants of LKB1 were transfected into HeLa cells using Lipofectamine 3000 (Invitrogen, Carlsbad, CA) following manufacturer’s guidelines. Cells were cultured for additional 48 h prior experiments. For total protein lysates, cells were lysed in radio-immunoprecipitation assay buffer (RIPA: 10 M Tris-HCl, pH 7.4, 150 mM NaCl, 0.1% sodium dodecyl sulfate [SDS], 1% Triton X-100, 1% sodium deoxycholate, 5 mM EDTA, 1 mM NaF, 1 mM sodium orthovanadate and protease inhibitors). Lysates were clarified by centrifugation at 13,000 rpm for 10 min, and protein concentrations were determined using a bicinchoninic acid assay kit (Pierce Chemical). Proteins (10 μg) were separated by SDS-polyacrylamide gel electrophoresis (SDS-PAGE) (8–12%) [38], transferred to polyvinylidene difluoride (PVDF) membranes and immunodetected using the indicated antibodies. Proteins were detected using enhanced chemiluminescence (Amersham Biosciences). Resulting immunoreactive bands were quantified using FluorChem Q Imaging software (Alpha Innotech).

### Subcellular fractionation

Following Zyflamend treatment, fractionation was performed in HeLa cells as described previously with modifications [39, 40]. Briefly, cells were washed with cold buffer A (100 mM sucrose, 1 mM EGTA, 20 mM 3-(N-morpholino)propanesulfonic acid (MOPS), pH 7.4) and resuspended in lysis buffer B (100 mM sucrose, 1 mM EGTA, 20 mM MOPS, 0.1 dithiothreitol (DTT), 5% freshly added percoll, 0.01% digitonin, 1 mM phenylmethylsulfonyl fluoride (PMSF) and cocktail of protease inhibitors, pH 7,4). Membranes were broken using a dounce homogenizer (200 strokes/sample). Debris and unbroken cells were removed by centrifugation (500 g for 10 min) and supernatants were then centrifuged (2500 g, 5 min) to separate nuclei (pellet). Supernatants were centrifuged again (15,000 g, 15 min) to separate mitochondria. Nuclear fraction was resuspended in radioimmunoprecipitation assay (RIPA) buffer containing proteases inhibitors. Cellular distribution and translocation of the indicated proteins were analyzed by SDS-PAGE and Western blot as described above. Purity of nuclear and cytoplasmic fractions was verified using antibodies against histone B and tubulin (Santa Cruz Biotechnology, Dallas, TX), respectively.

### Western blotting

Cells were lysed in RIPA lysis buffer (Thermo Scientific, Rockford, IL). Protein concentration was measured using a Bradford protein assay (Thermo Scientific, Rockford, IL). Equal amount of protein (30 μg) were separated by 8% SDS-PAGE and transferred to a PVDF membrane by electroblotting. Membranes were blocked by 5% non-fat dry milk (LabScientific, Highlands, NJ) or bovine serum albumin (Santa Cruz Biotechnology, Dallas, TX) in 0.1% Tris-buffered saline-Tween-20 (TBST) for 1 h at room temperature and incubated in TBST containing primary antibodies overnight at 4 °C. Membranes were incubated with anti-rabbit or anti-mouse secondary antibody conjugated with horseradish peroxidase (HRP) (Cell Signaling Technology, Danver, MA) for 1 h at room temperature. Protein expression was detected with Super Signal West Pico Chemiluminescent Substrate (Thermo Scientific, Rockford, IL) and membranes were exposed and analyzed via *Li-Cor Odyssey FC* imaging system (Li-Cor, Lincoln, NE). Antibodies against p-AMPKα (Thr172), AMPKα, p-ACC (Ser79), ACC, p-LKB1 (Ser428), LKB1, p- PKCζ (Thr410), PKCζ, and p-CaMKKβ (Ser511) were used to detect target protein level at 1:1000. β-Actin or glyceraldehyde 3-phosphate dehydrogenase (GAPDH) (Santa Cruz Biotechnology, Dallas, TX) was used as the loading control.

### ATP assay

Cellular ATP concentration was determined using a fluorometric ATP assay kit (BioRad, Milpitas, CA) following the manufacturer’s instructions, and fluorescence was read at 525 nm on a Glowmax Multi Detection System (Promega Corporation, Madison, WI).

### Statistics

For Western blot, protein was analyzed from 3 independent samples and presented as mean ± SEM. For ATP concentration, results are presented as mean ± SEM. For multiple comparisons, data was analyzed using IBM SPSS Statistics 24 and tested by one-way analysis of variance (ANOVA) followed by a Tukey’s post-hoc test. Two-group comparisons were analyzed by two-tailed Student’s T-test. Results were considered statistically significant at *p* < 0.05.

## Results

### Effect of Zyflamend on cell proliferation, ATP levels and AMPK phosphorylation in CWR22Rv1 cells

**Fig. 2:**
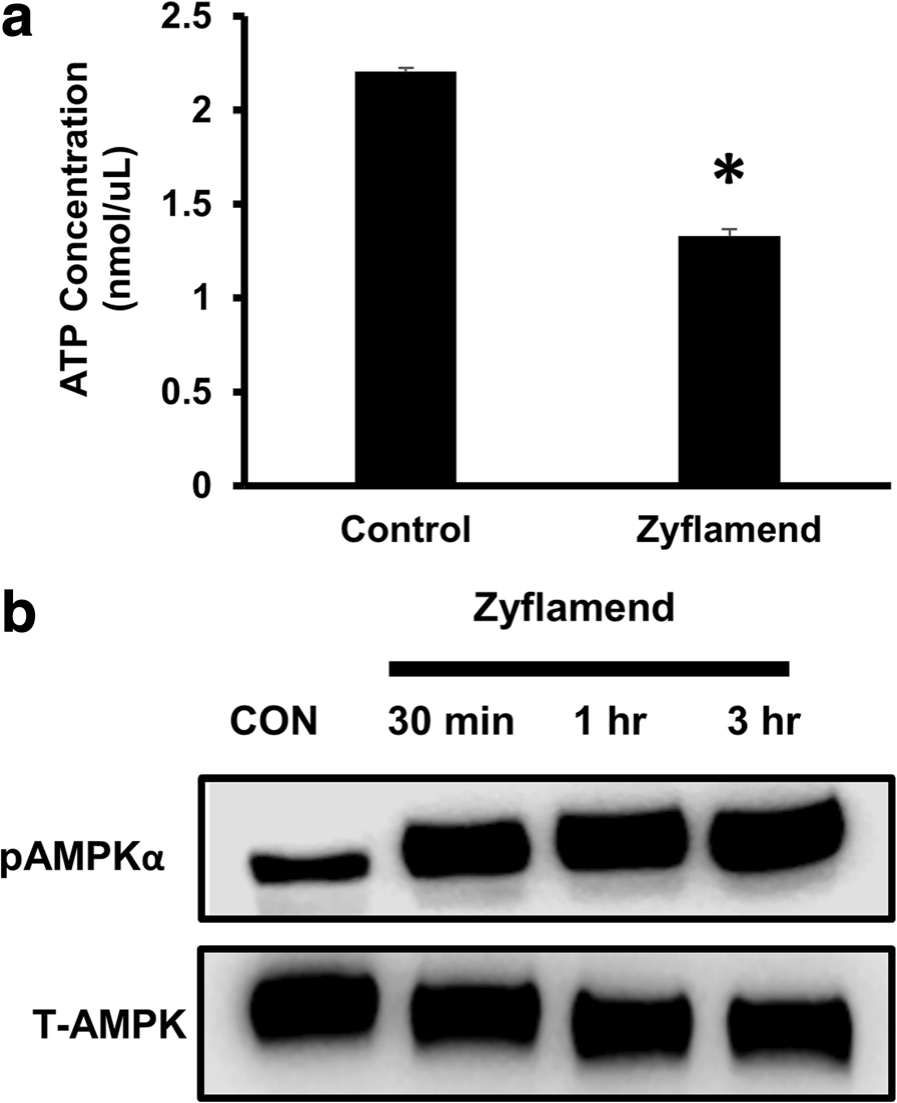
The effects of Zyflamend on cellular ATP levels and phosphorylation of AMPKα (at Thr172) in CWR22Rv1 cells. **a** ATP levels of CWR22Rv1 cells treated in the presence or absence of Zyflamend (200 μg/mL, 30 min). **b** The effects of Zyflamend (200 μg/mL, 30 min – 3 h) on phosphorylation of AMPKα in CWR22Rv1 cells. Data is presented as mean ± SEM, *n* = 4. Abbreviations: Con, Control

Zyflamend (200 μg/mL) inhibited cell proliferation in CWR22Rv1 cells in a concentration and time dependent manner (Additional file 1: Figure S1, replicating previously published results [6, 8]). Similar results were replicated in a variety of immortalized prostate-derived cells lines [8] and in the HCT116 colorectal cell line (Additional file 2: Figure S2). Because Zyflamend has been shown to change the energetics of CWR22Rv1 cells [3, 10], levels of ATP were determined. In the presence of Zyflamend, ATP levels were reduced by ~ 40% (Fig. 2a) and AMPK phosphorylation (Thr172) was significantly increased (Fig. 2b). These results were replicated in the HCT116 colorectal cell line to demonstrate that these effects are not specific for prostate cancer cells (Additional file 2: Figure S2).

### AMPK activation by CaMKK2 in the presence and absence of Zyflamend in CWR22Rv1 cells

**Fig. 3:**
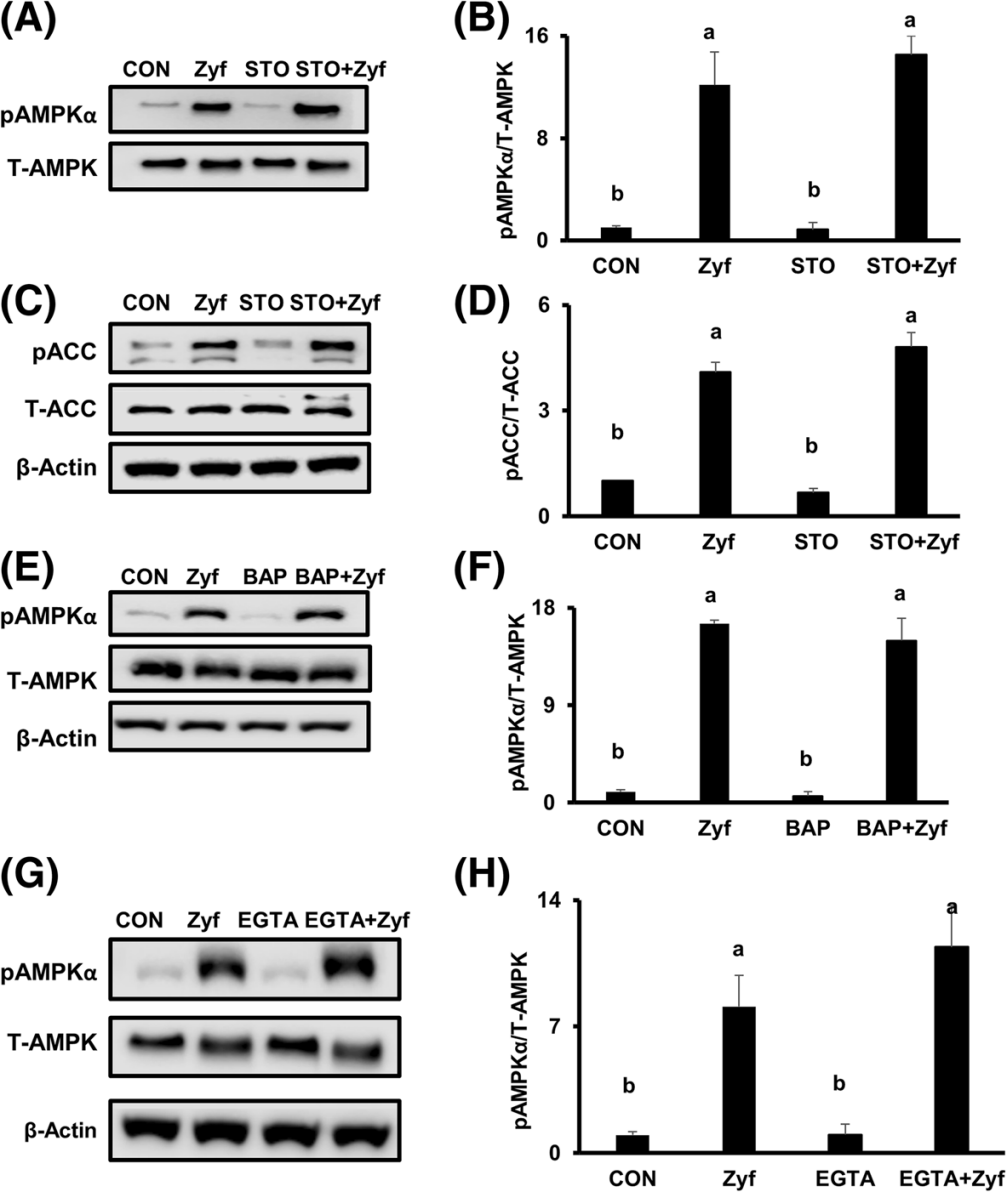
The effects of CaMKK2 inhibition by STO-609, BAPTA-AM and EGTA on pAMPKα (at Thr172) and pACC (at Ser79), ± Zyflamend in CWR22Rv1 cells. (**A**-**D**) Western blot of pAMPKα and pACC following treatment of STO-609 (10 μM, 30 min) ± Zyflamend (200 μg/mL, 30 min). (**E**, **F**) Western blot of pAMPKα following treatment of BAPTA-AM (30 μM, 30 min) ± Zyflamend (200 μg/mL, 30 min). (**G**, **H**) Western blot of pAMPKα following treatment of EGTA (2 mM, 30 min) ± Zyflamend (200 μg/mL, 30 min). Data are presented as mean ± SEM, *n* = 3. Bars with different letters are statistically different at *p* < 0.05. Abbreviations: BAP, BAPTA-AM (1, 2-bis(*o*-aminophenoxy)ethane-*N*,*N*,*N′*,*N′*-tetraacetic acid acetoxymethyl ester); CON, Control; EGTA, ethylene glycol-bis (β-aminoethyl ether)-N, N, N′,N′-tetraacetic acid; STO, STO-609; Zyf, Zyflamend

**Fig. 4:**
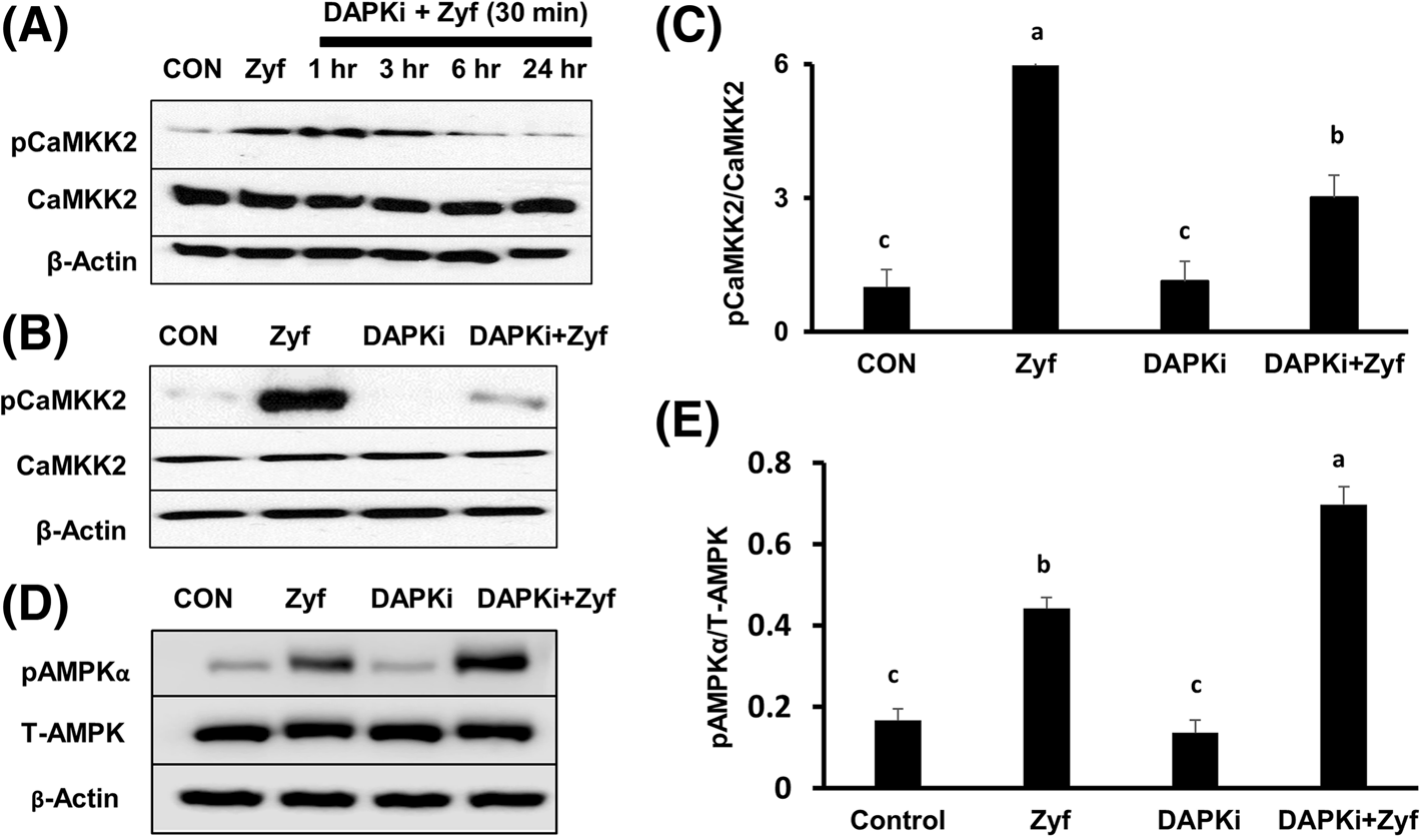
The effects of Zyflamend and an inhibitor of DAPK on the phosphorylation of CaMKK2 (at Ser511), in CWR22Rv1 cells. **A** Western blot of pCaMKK2 ± Zyflamend (200 μg/mL, 30 min) and following treatment of DAPKi (20 μM, 1 h – 24 h) followed by the treatment of Zyflamend (200 μg/mL, 30 min). **B**, **C** Western blot and graph comparison of pCaMKK2 following treatment of DAPKi (20 μM, 24 h) ± Zyflamend (200 μg/mL, 30 min). (**D**, **E**) Western blot and graph comparison of pAMPK following treatment of Zyflamend (200 μg/mL, 30 min) and DAPKi (20 μM, 24 h) ± Zyflamend (200 μg/mL, 30 min). Data are presented as mean ± SEM, *n* = 3. Bars with different letters are statistically different at *p* < 0.05. Abbreviations: CON, Control; DAPKi, DAPK inhibitor; Zyf, Zyflamend

Zyflamend significantly increased the phosphorylation of AMPK (Thr172) (Fig. 3A and B) and its downstream target ACC (Ser79) (Fig. 3C and D), results unaffected by pretreatment with the CaMKK2 inhibitor STO-609 (Fig. 3A-D, lane 4/bar 4). To confirm that the activation of AMPK by Zyflamend is independent of CaMKK2, cells were pre-treated with the calcium chelators BAPTA-AM and EGTA, as CaMKK2 activation is dependent upon intracellular calcium. Pretreatment with BAPTA-AM (Fig. 3E and F) and EGTA (Fig. 3G and H) failed to prevent phosphorylation of AMPK in the presence of Zyflamend. Zyflamend increased the phosphorylation of CaMKK2 at Ser511 (Fig. 4A), a phosphorylation site that results in its inhibition and reported to be mediated by DAPK [37]. Pretreatment with a DAPK inhibitor attenuated Zyflamend’s ability to increase phosphorylation of CaMKK2 at Ser511 in a time-dependent manner (Fig. 4A-C), suggesting phosphorylation of CaMKK2 in the presence of Zyflamend is mediated by DAPK. Pretreatment of cells with DAPKi failed to prevent phosphorylation of AMPK in the presence of Zyflamend, further confirming that the activation of AMPK by Zyflamend is independent of CaMKK2 (Fig. 4D and E).

### AMPK activation by LKB1 in the presence and absence of Zyflamend in CWR22Rv1 cells

**Fig. 5:**
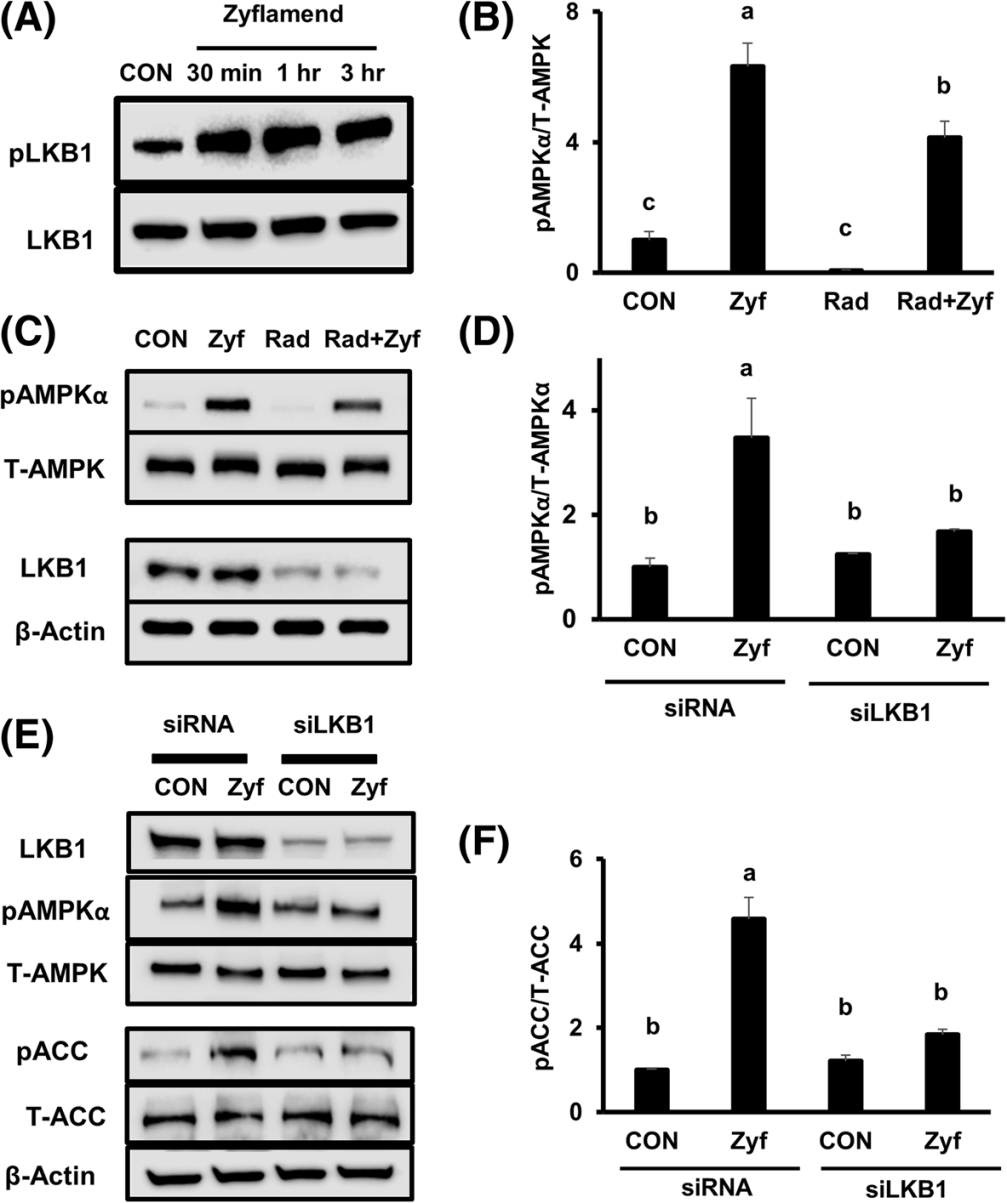
The effects of Zyflamend on phosphorylation of LKB1 (at Ser428) and AMPKα (at Thr172) following inhibition with radicicol and knockdown of LKB1 in CWR22Rv1 cells. **A** Western blot of pLKB1 ± Zyflamend (200 μg/mL, 30 min – 3 h). **B**, **C** Western blot of pAMPKα following treatment with radicicol (5 μM, 24 h) ± Zyflamend (200 μg/mL, 30 min). **D**-**F** Western blot of pAMPKα and pACC (at Ser79) following knockdown of LKB1 ± Zyflamend (200 μg/mL, 30 min). Data are presented as mean ± SEM, *n* = 3. Bars with different letters are statistically different at *p* < 0.05. Abbreviations: CON, Control; Rad, Radicicol; Zyf, Zyflamend

Zyflamend significantly increased the phosphorylation of LKB1 (Ser428) (Fig. 5A), AMPK (Thr172) (Fig. 5C) and ACC (Ser79) (Fig. 5E). In the presence of radicicol, a non-specific inhibitor of LKB1 that results in reduction of total LKB1 protein levels (Fig. 5C, row 3, column 3), phosphorylation of AMPK failed to be fully restored upon Zyflamend treatment (Fig. 5B and C). Likewise, knockdown of LKB1 by siRNA (Fig. 5d-f) inhibited Zyflamend-induced phosphorylation of AMPK (Fig. 5D and E) and its downstream target ACC (Fig. 5E and F).

### Zyflamend-induced AMPK phosphorylation is LKB1 dependent

**Fig. 6:**
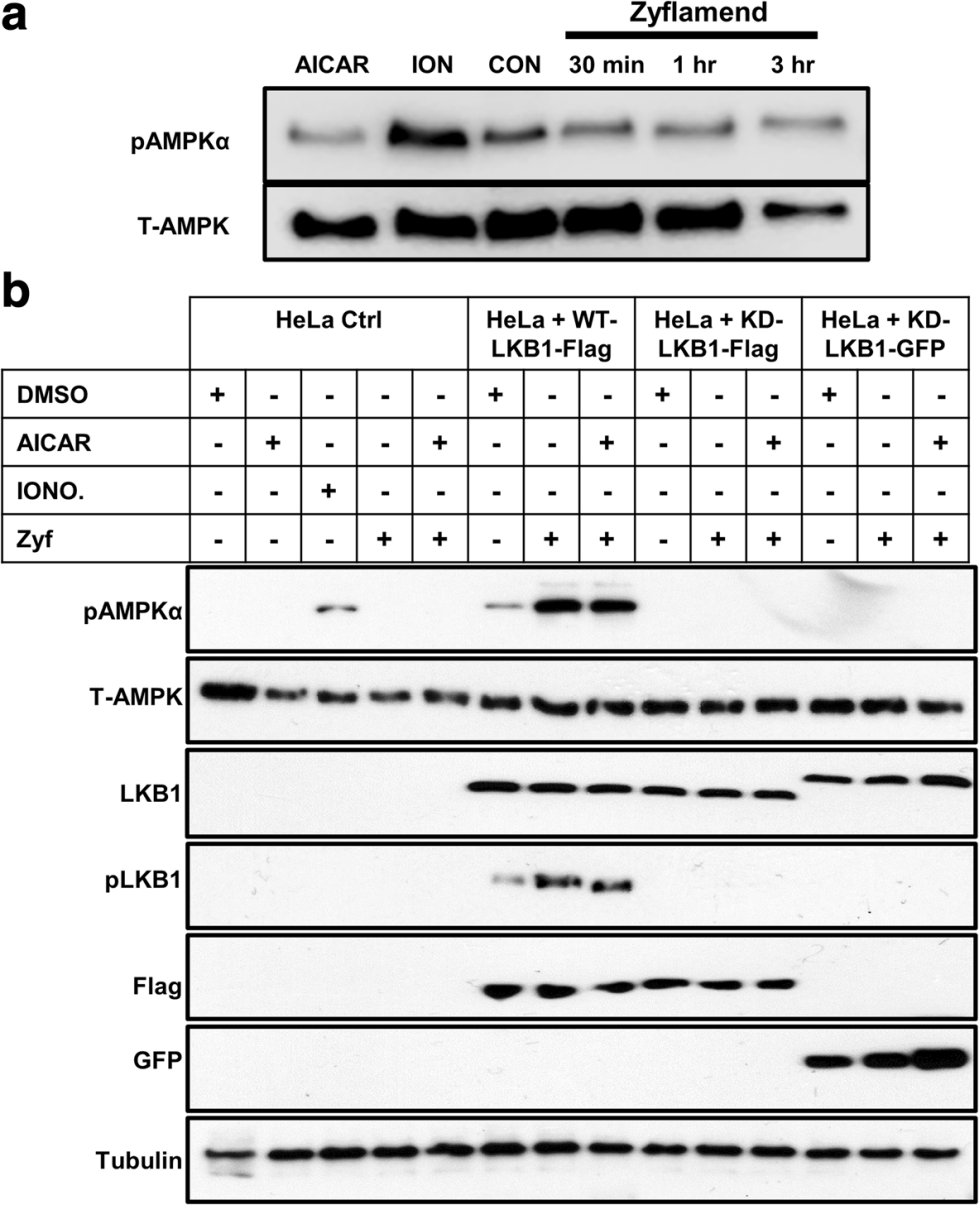
Effects of Zyflamend on pAMPK (at Thr172) and pLKB1 (at Ser428) in HeLa cells null for LKB1, transfected with wild type (WT) LKB1 or with two catalytically dead (KD) mutants of LKB1. **a** Western blot of pAMPK in HeLa cells ± AICAR, (1 mM, 1 h), ionomycin (1 μM, 1 h) or Zyflamend (200 μg/mL, 30 min – 3 h). (**b**) Western blot of pAMPK in HeLa cells, WT-LKB1 HeLa cells, and KD-LKB1 HeLa cells ± AICAR (1 mM, 1 h), ionomycin (1 μM, 1 h), and/or Zyflamend (200 μg/mL, 1 h). Representative immunoblots from 3 independent experiments are shown. Abbreviations: Con, Control; DMSO, dimethyl sulfoxide; Ion, ionomycin; Zyf, Zyflamend

To confirm that Zyflamend-induced phosphorylation of AMPK is LKB1 dependent and CaMKK2 independent, we treated LKB1-null HeLa cells, that constitutively express CaMKK2, with AICAR (an activator of AMPK that is commonly used as a positive control), ionomycin (activator of CaMKK2) and Zyflamend (Fig. 6a). The same experiment was conducted on HeLa cells that were stably transfected with the wild-type (WT) or the catalytically dead (KD) mutants of the human LKB1. Two different constructs of the KD mutants were used, Flag-tagged or green fluorescent protein (KD-LKB1 Flag or KD-LKB1 GFP, respectively) (Fig. 6b). In both mutants, lysine 78 was mutated to isoleucine, abolishing auto-phosphorylation and activation of LKB1 [41, 42]. Ionomycin (Fig. 6b, column 3), but not AICAR (column 2) or Zyflamend (column 4), induced phosphorylation of AMPK in LKB1-null HeLa cells (control) (Fig. 6b, columns 1–5). Following transfection with WT-LKB1 (Fig. 6b, columns 6–8), Zyflamend induced phosphorylation of LKB1 and AMPK (column 7), an effect more pronounced with co-treatment of AICAR (column 8). However, no phosphorylation of LKB1 and AMPK was observed in the KD mutants following treatment with Zyflamend and Zyflamend+AICAR (Fig. 6b, columns 9–14).

### Zyflamend-mediated LKB1 phosphorylation is linked to PKCzeta

**Fig. 7:**
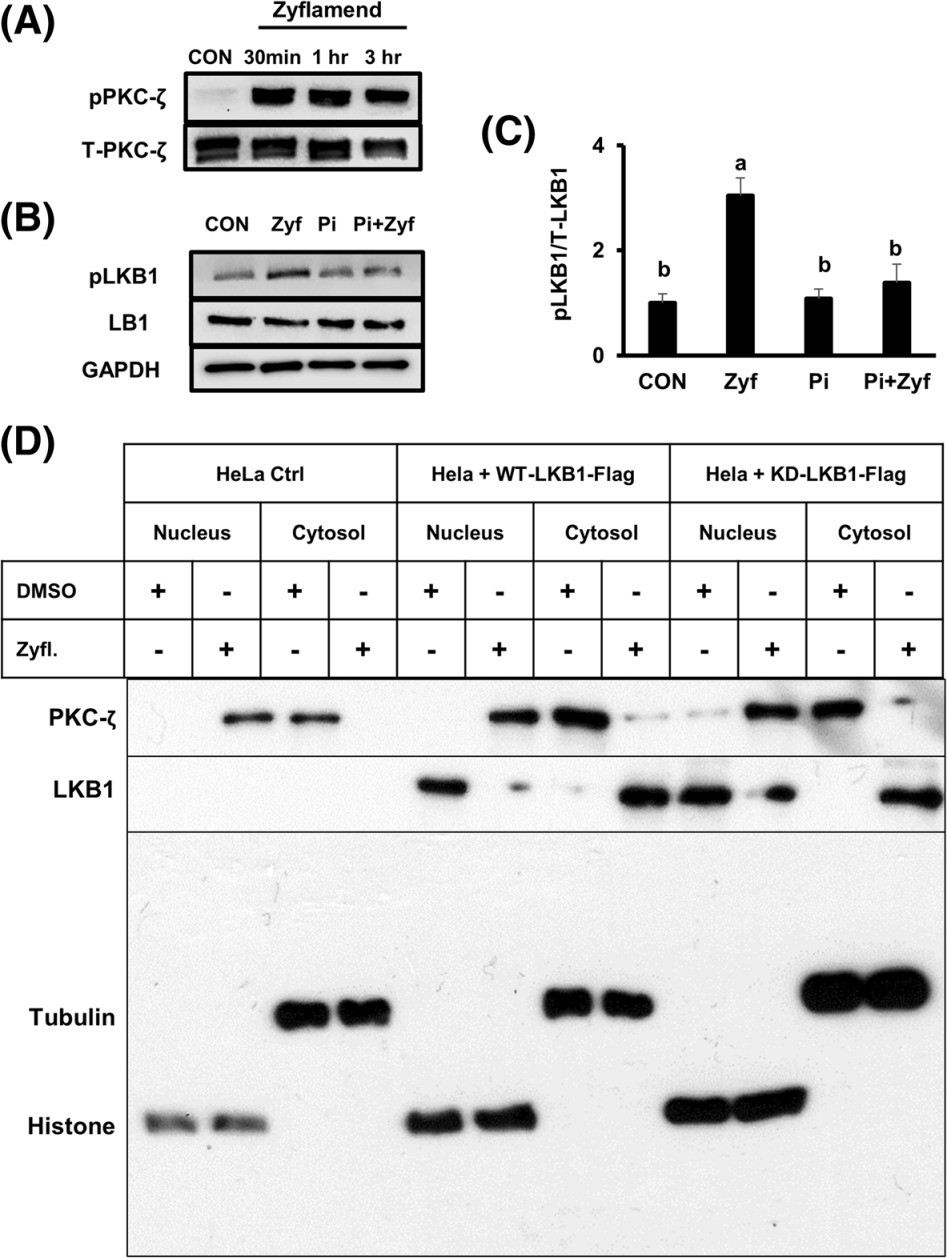
The effects of Zyflamend on phosphorylation of PKCζ (at Thr410) and cellular location of PKCζ and LKB1 in HeLa cells null for LKB1, transfected with wild type (WT) LKB1 or with a catalytically dead (KD) mutant of LKB1. **A** Western blot of phosphorylation of PKCζ ± Zyflamend (200 μg/mL, 30 min – 3 h). **B**, **C** Western blot of pLKB1 (at Ser428) following treatment with Zyflamend, PKCζ inhibitor (5 μM, 30 min) or pretreatment with the PKCζ inhibitor plus Zyflamend (200 μg/mL, 30 min). **D** Western blot for PKCζ and LKB1 following subcellular fractionation (cytosol and nucleus) in HeLa cells null for LKB1, transfected with WT LKB1 or with a KD mutant of LKB1 treated with or without Zyflamend (200 μg/mL, 1 h). Representative immunoblots from 3 independent experiments are shown. Abbreviations: CON, Control; Pi, PKCζ inhibitor; Zyf, Zyflamend

In an effort to determine how Zyflamend may be mediating the phosphorylation of LKB1, PKCζ was investigated as a possible upstream target (Fig. 7). Phosphorylation of PKCζ and LKB1 increased when CWR22Rv1 cells were treated with Zyflamend (Fig. 7A). However, in the presence of a highly selective PKCζ pseudo-substrate inhibitor, phosphorylation of LKB1 could not be restored to control levels following Zyflamend treatment (Fig. 7B and C). Using our informative HeLa cell constructs, null for LKB1 and transfected with WT-LKB1 or KD-LKB1, we further investigated the relationship between PKCζ and LKB1 (Fig. 7D). In HeLa cells devoid of LKB1 (control cells), PKCζ is located in the cytosol (Fig. 7D, row 1, column 3), but appears to translocate to the nucleus upon treatment with Zyflamend (Fig. 7D, row 1, column 2). In cells transfected with WT-LKB1, PKCζ is located in the cytosol (Fig. 7D, row 1, column 7) and LKB1 is located in the nucleus (Fig. 7D, row 2, column 5). Following Zyflamend treatment, their locations switch, where PKCζ translocates to the nucleus (Fig. 7D, row 1, column 6) and LKB1 is found in the cytosol (Fig. 7D, row 2, column 8). This translocation following Zyflamend treatment appears to be independent of a catalytically active protein, as the same results were observed with the KD-LKB1 mutant (Fig. 7D, columns 9–12).

## Discussion

Zyflamend is a unique blend of ten herbal extracts with tumor suppressor properties whose biochemical and physiological effects have been replicated in different laboratories, at different times, using different lots, with similar doses/concentrations [6–8, 10, 11, 13–17, 19, 21, 43]. The quality control of this preparation has been summarized elsewhere [7] and is most likely responsible for the reproducibility of results. Importantly, the effects of Zyflamend have been reported in a variety of cell lines, not just prostate cancer cells (CWR22, CWR22R, CWR22Rv1, PC3, LNCaP, RWPE-1, RAW 264.7, H1299, KBM-5, U266, MeW0, A365, MSK-Leuk1, HaCaT, HCT116, THP-1, HEK293) [6–8, 10–19, 21, 43]. While there is clinical evidence for the beneficial effects of Zyflamend on prostate cancer [9, 18, 20, 44], it is not possible to tease out the contributions and/or interactions of each constituent due to the high number of possible combinations (as many as 1024). However, recent studies have demonstrated that combining components from this blend enhances their cellular and molecular effects by orders of magnitude as compared to isolated components, indicating highly synergistic interactions when combined [22]. The ability of this mixture to function at human equivalent doses and in clinical trials is most likely due to this synergy. This combination has been shown to be clinically effective in prostate cancer patients by reducing levels of prostate specific antigen (PSA), a biomarker used to monitor prostate cancer progression. Case studies from M.D. Anderson Cancer Center report dramatic reductions in PSA levels following treatment with Zyflamend and/or metformin (an activator of AMPK) in patients whose prostate cancer no longer responds to a variety of standard therapies [9], an effect also observed in our recent clinical trial with prostate cancer patients undergoing radical prostatectomy [44].

Castrate-resistant prostate cancer is the focus of this research and the research in our laboratory. To study mechanisms of action, we use human prostate cancer cells derived from the CWR22 lineage [6–8, 10, 45, 46]. Similar to the progression of human prostate cancer, these cells are originally androgen dependent and can transform to a castrate-resistant line in vivo (i.e., CRW22R) following hormone ablation [46–48]. Unlike some other prostate cancer cell lines (i.e., PC3 cells), the CWR22Rv1 cells express a constitutively active androgen receptor and PSA, characteristics shared by castrate-resistant prostate cancer in humans.

The effectiveness of Zyflamend on prostate cancer rests, in part, with its ability to upregulate AMPK, an effect observed in other cell types and tissues (i.e., HCT116, PC3, adipose) suggesting a general effect [10, 44]. However, the mechanism as to how Zyflamend upregulates AMPK is unknown, although many of its constituents have been shown to independently activate AMPK by modifying mitochondrial ATP production (as reviewed in [49]). This is the first paper to delineate the coordination of potential upstream pathways involved in the activation of AMPK, viz., LKB1 and CaMKK2, and to do so using natural products.

The role of AMPK in prostate cancer is controversial in the sense that upstream kinases responsible for its activation appear to have contradictory effects on cancer [29]. CaMKK2 is a known tumor promotor whose expression is linked to the upregulation of the androgen receptor, a key step in castrate-resistant prostate cancer [4, 31]. Interestingly, Zyflamend down regulates the androgen receptor and its nuclear localization [6]. DAPK is a differentially methylated gene where most of its effects on cancer have focused on its epigenetic regulation [33–36]. Uniquely, a catalytic downstream target of DAPK is CaMKK2 where it phosphorylates CaMKK2 at Ser511 [37], a site adjacent to the Ca^+ 2^-calmodulin regulatory domain, preventing autophosphorylation and inhibiting catalytic activity [37].

In contrast, LKB1 exhibits tumor suppressor properties, where loss of LKB1 is involved in a variety of cancers [24, 30]. LKB1-mediated activation of AMPK is dependent upon increases in the AMP(ADP):ATP ratios. Zyflamend significantly decreases ATP in the cells. LKB1 contains a nuclear localization domain and is typically (but not always exclusively) found in the nucleus. Following activation, LKB1 co-localizes with STE20-related adaptor (known as STRAD) protein and scaffolding mouse 25 (known as MO25) protein and translocates to the cytosol where it exerts its kinase activity on a number of downstream targets, including AMPK [50]. Nuclear export, in part, appears to involve phosphorylation at Ser428 by PKCζ [51].

**Fig. 8:**
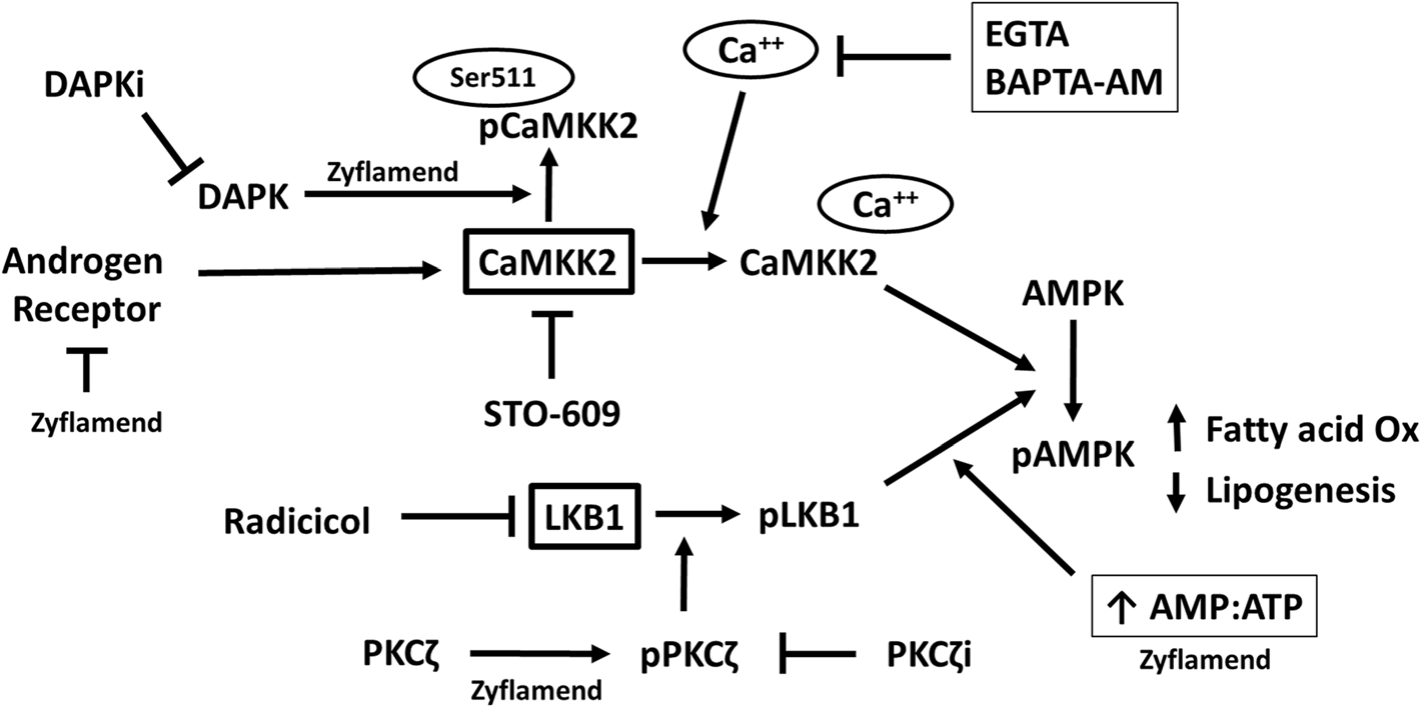
Summary of the effects of Zyflamend on AMPK regulation by signaling pathways of LKB1 and CaMKK2. Zyflamend has been shown to inhibit castrate-resistant prostate cancer, in part, through the activation of AMPK. This activation is mediated by the increase in AMP:ATP ratio and the activation of the tumor suppressor protein LKB1 following phosphorylation by PKCζ. Simultaneously, Zyflamend inhibits the tumor promotor CaMKK2 via phosphorylation at Ser511 by DAPK

A key finding from this research is that Zyflamend antithetically regulates two parallel pathways important in the phosphorylation of AMPK that is potentially important in castrate-resistant prostate cancer. These effects are summarized in Fig. 8. Zyflamend-mediated activation of AMPK appears to be LKB1 dependent, while coordinately and negatively regulating CaMKK2 activity. This was observed using LKB1-null and KD-LKB1 transfected HeLa cells that constitutively express CaMKK2. The addition of ionomycin (activator of CaMKK2) robustly increased phosphorylation of AMPK, but Zyflamend (with and without AICAR, an AMP analog) had no effect. Our results suggest that Zyflamend inhibits CaMKK2 following DAPK-mediated phosphorylation at Ser511, as this effect is prevented by the presence of a DAPK inhibitor.

On the other hand, Zyflamend robustly increased the phosphorylation of AMPK only in HeLa cells transfected with WT LKB1. Using the various constructs of the HeLa cells, we confirmed nuclear localization of LKB1, with translocation to the cytosol following Zyflamend treatment (Fig. 7). Zyflamend increased phosphorylation of PKCζ, a known activator of LKB1, and inhibition of PKCζ reduced LKB1 phosphorylation in the presence of Zyflamend. Importantly, translocation of PKCζ from the cytosol to the nucleus occurred concomitantly.

These results help explain why inhibitors of CaMKK2 (STO-609, BAPTA-AM, EGTA, DAPKi) failed to prevent the activation of AMPK in the presence of Zyflamend in CWR22Rv1 cells. This was due to the simultaneous activation of LKB1, and this was confirmed when activation of AMPK (in the presence of Zyflamend) was not completely rescued following inhibition (radicicol) and knockdown (siRNA) of LKB1.

## Conclusions

In summary, Zyflamend has been shown to inhibit castrate-resistant prostate cancer, in part, through the activation of AMPK, results confirmed using chemical and molecular interventions [10]. In conjunction with reducing ATP levels, this activation is mediated by the tumor suppressor protein LKB1, via activation of PKCζ. Simultaneously, Zyflamend inhibits CaMKK2, a tumor promotor that is over-expressed in many cancers, including castrate-resistant prostate cancer. This inhibition appears to be uniquely mediated by DAPK. More studies are warranted to interrogate the relationship of Zyflamend with DAPK signaling. In conclusion, this is the first evidence that multiple upstream pathways involved in the activation of AMPK, an important signaling molecule in cancer, can be simultaneously regulated using a well-defined natural product.

## Additional files

Additional file 1: **Figure S1.** Effect of Zyflamend on the proliferation of a castrate resistant prostate cancer cells in vitro. CWR22Rv1 cells were treated with Zyflamend (0–200 μg/ml) from 0 to 96 h and cell proliferation was monitored using the MTT assay. (PDF 71 kb)

Additional file 2: **Figure S2.** Effect of Zyflamend on the proliferation of a colorectal cancer cell line in vitro and the subsequent phosphorylation of AMPKα at Thr172. (A) HCT116 cells were treated with Zyflamend (0–200 μg/ml) from 0 to 72 h and cell proliferation was monitored using the MTT assay. (B) Phosphorylation of AMPKα at Thr172 was determined following Zyflamend treatment (200 μg/ml for 3 h). (PDF 254 kb)

## Abbreviations

ACC: Acetyl CoA carboxylase
AICAR: 5-aminoimidazole-4-carboxamide ribonucleotide
AMPK: 5′-adenosine monophosphate-activated protein kinase
ANOVA: Analysis of variance
BAPTA-AM: 1,2-bis(*o*-aminophenoxy)ethane-*N*,*N*,*N′*,*N′*-tetraacetic acid acetoxymethyl ester
CaMKK,2: Calcium-calmodulin kinase kinase-2
CR-PCa: Castrate-resistant prostate cancer
DAPK: Death-associated protein kinase Inhibitor
DTT: Dithiothreitol
EGTA: Ethylene glycol-bis(β-aminoethyl ether)-N,N,N’,N’-tetraacetic acid
GAPDH: Glyceraldehyde 3-phosphate dehydrogenase
GFP: Green fluorescent protein
LKB1: Liver kinases B1
MLK3: Mixed lineage kinase 3
MO25: Scaffolding mouse 25 protein
MOPS: 3-(N-morpholino)propanesulfonic acid
PKCζ: Protein kinase C-zeta
PMSF: Phenylmethylsulfonyl fluoride
RIPA: Radioimmunoprecipitation assay
STRAD: STE20-related adaptor protein
TAK1: Transforming growth factor-β activated protein kinase-1
