# Supplementary material for: Concurrent regulation of LKB1 and CaMKK2 in the activation of AMPK in castrate-resistant prostate cancer by a well-defined polyherbal mixture with anticancer properties
Source: BMC Complement Altern Med. 2018 Jun 18;18:188. doi: 10.1186/s12906-018-2255-0 (PMC6006779; doi:10.1186/s12906-018-2255-0)
Supplement: Supplementary file 1 — Figure S1. Effect of Zyflamend on the proliferation of a castrate resistant prostate cancer cells in vitro. CWR22Rv1 cells were treated with Zyflamend (0–200 μg/ml) from 0 to 96 h and cell proliferation was monitored using the MTT assay. (PDF 71 kb) [file 12906_2018_2255_MOESM1_ESM.pdf]

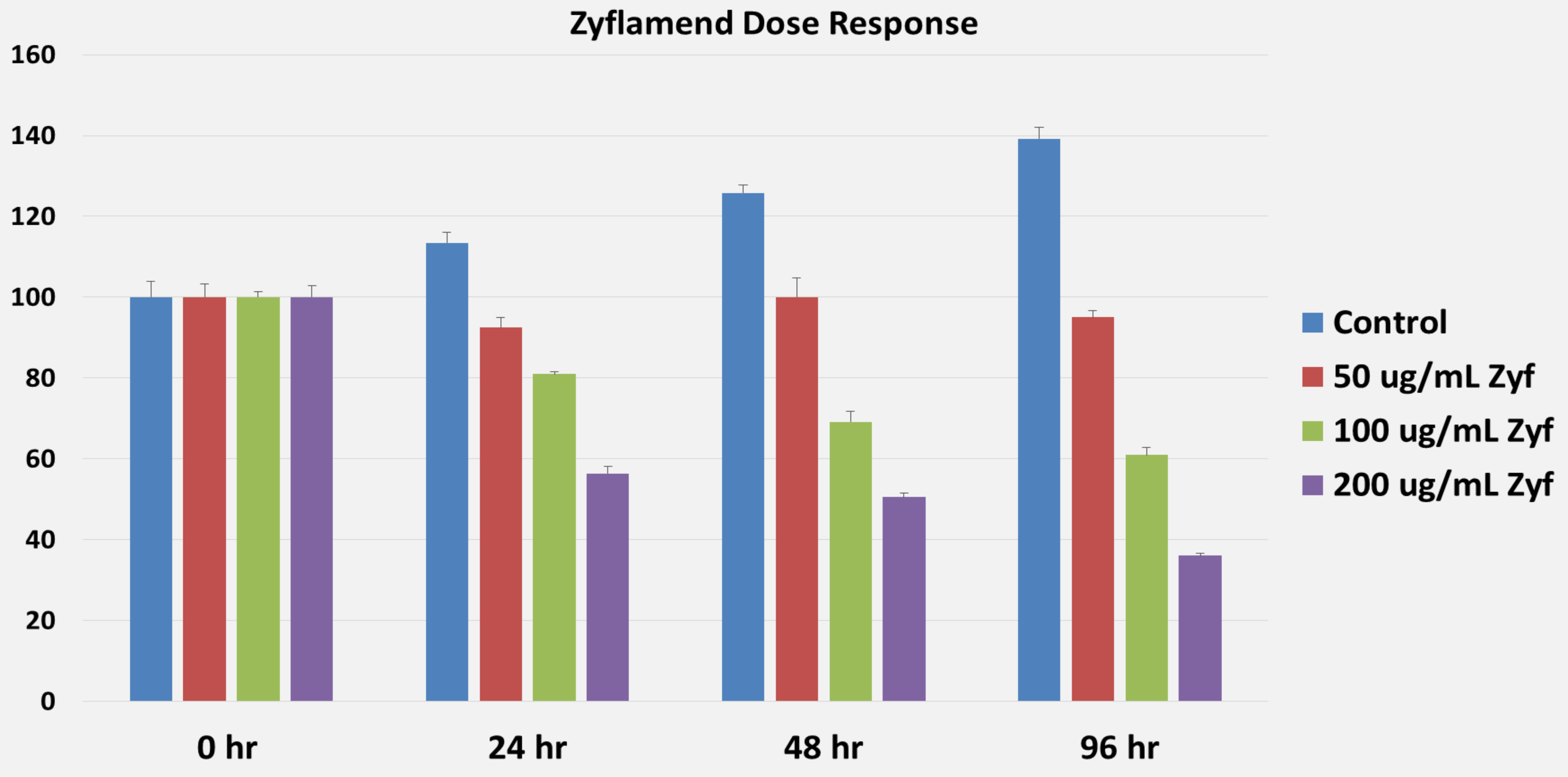

Figure 1: Effect of Cell Proliferation ± Zyflamend in CWR22Rv1s.
Graph of MTT assay ± Zyflamend (50 ug/mL, 100 ug/mL, 200 ug/mL, 0-96 hr). Error Bars are SEM; n=8.
