# Supplementary material for: Concurrent regulation of LKB1 and CaMKK2 in the activation of AMPK in castrate-resistant prostate cancer by a well-defined polyherbal mixture with anticancer properties
Source: BMC Complement Altern Med. 2018 Jun 18;18:188. doi: 10.1186/s12906-018-2255-0 (PMC6006779; doi:10.1186/s12906-018-2255-0)
Supplement: Supplementary file 2 — Figure S2. Effect of Zyflamend on the proliferation of a colorectal cancer cell line in vitro and the subsequent phosphorylation of AMPKα at Thr172. (A) HCT116 cells were treated with Zyflamend (0–200 μg/ml) from 0 to 72 h and cell proliferation was monitored using the MTT assay. (B) Phosphorylation of AMPKα at Thr172 was determined following Zyflamend treatment (200 μg/ml for 3 h). (PDF 254 kb) [file 12906_2018_2255_MOESM2_ESM.pdf]

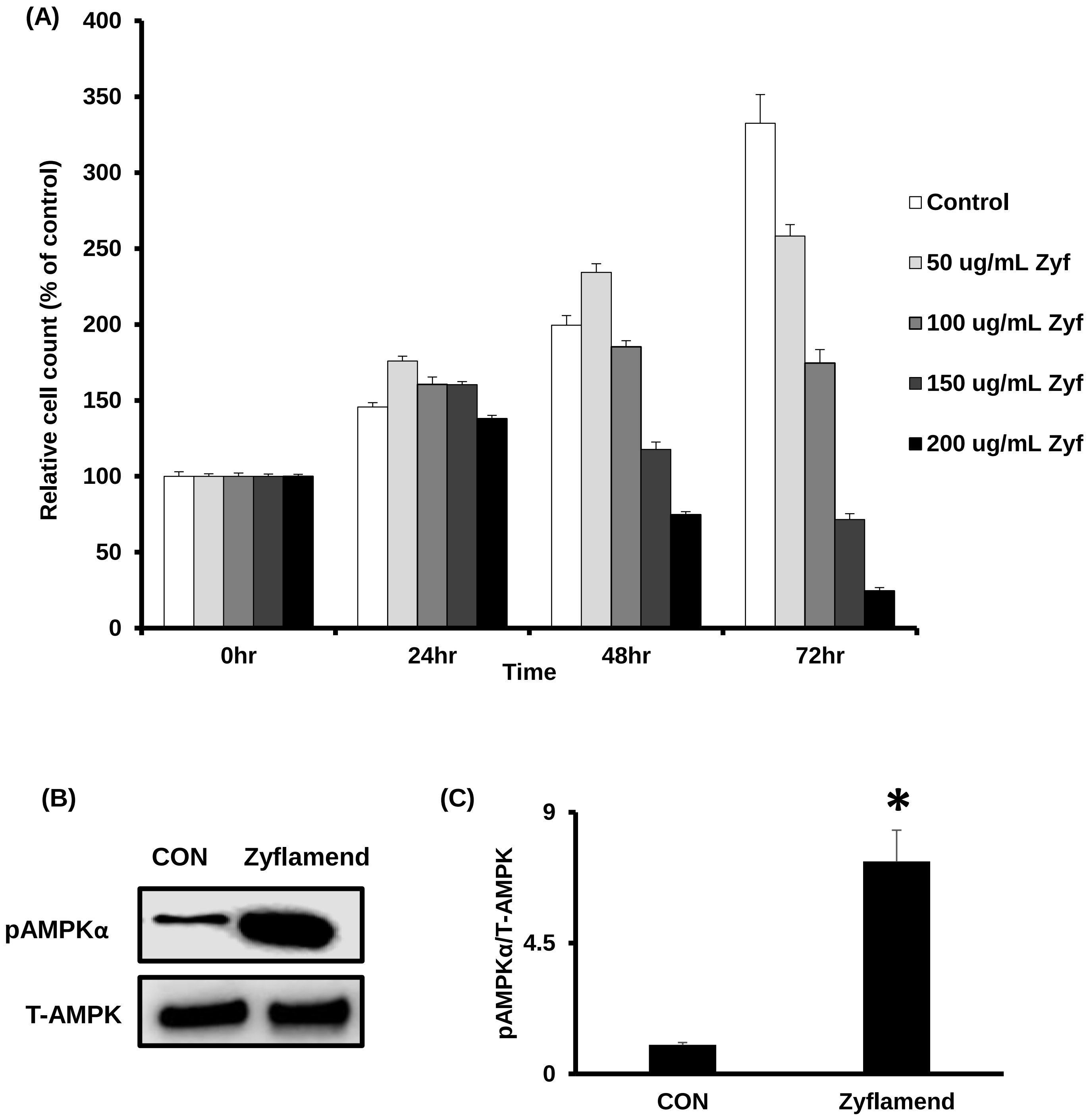

Supplemental Figure 2. The effects of Zyflamend on cell proliferation and phosphorylation of AMPKα (at Thr172) in HCT 116 cells. (A) MTT assay of HCT 116 cells in the presence or absence of Zyflamend (50-200 μg/mL, 0 hr – 72 hr). (B, C) The effects of Zyflamend (200 μg/mL, 3 hr) on phosphorylation of AMPKα in HCT 116 cells. Data is presented as mean ± SEM, n=8 (A) and n=3 (C) and statistically significant at $p<0.05$. Abbreviations: Con, Control.
